# Metabolomic Biomarker Identification in Presence of Outliers and Missing Values

**DOI:** 10.1155/2017/2437608

**Published:** 2017-02-14

**Authors:** Nishith Kumar, Md. Aminul Hoque, Md. Shahjaman, S. M. Shahinul Islam, Md. Nurul Haque Mollah

**Affiliations:** ^1^Bioinformatics Lab, Department of Statistics, Rajshahi University, Rajshahi, Bangladesh; ^2^Department of Statistics, Bangabandhu Sheikh Mujibur Rahman Science and Technology University, Gopalganj, Bangladesh; ^3^Department of Statistics, Begum Rokeya University, Rangpur, Bangladesh; ^4^Institute of Biological Sciences, Rajshahi University, Rajshahi, Bangladesh

## Abstract

Metabolomics is the sophisticated and high-throughput technology based on the entire set of metabolites which is known as the connector between genotypes and phenotypes. For any phenotypic changes, potential metabolite (biomarker) identification is very important because it provides diagnostic as well as prognostic markers and can help to develop new biomolecular therapy. Biomarker identification from metabolomics data analysis is hampered by the use of high-throughput technology that provides high dimensional data matrix which contains missing values as well as outliers. However, missing value imputation and outliers handling techniques play important role in identifying biomarker correctly. Although several missing value imputation techniques are available, outliers deteriorate the accuracy of imputation as well as the accuracy of biomarker identification. Therefore, in this paper we have proposed a new biomarker identification technique combining the groupwise robust singular value decomposition, *t*-test, and fold-change approach that can identify biomarkers more correctly from metabolomics dataset. We have also compared the performance of the proposed technique with those of other traditional techniques for biomarker identification using both simulated and real data analysis in absence and presence of outliers. Using our proposed method in hepatocellular carcinoma (HCC) dataset, we have also identified the four upregulated and two downregulated metabolites as potential metabolomic biomarkers for HCC disease.

## 1. Introduction

Biomarker discovery is comparatively new and one of the most dominating fields of biological research. Different disciplines are involved with the study of biomarkers: clinical and environmental chemistry, molecular biology, toxicology, food research, plant and animal biology, and so on. In general, biomarkers identification research is needed whenever a whole set/pool set of features are differentiated into two or more groups of samples. In the area of metabolomics, biomarkers are small molecules (metabolites) that can be used for early warning indicators of disease, observing disease progression, and predicting receptivity to treatment. Thus, biomarkers may be categorized into three most important groups: diagnostic, prognostic, and predictive markers [1]. Diagnostic markers are required for early and/or accurate diagnosis of disease to allow best possible treatment choices. On the other hand, prognostic markers give information about future route of a disease that would influence the treatment choices. Predictive markers provide sagacity on the potential responses of an individual to the different treatment options. Therefore, biomarker discovery is very important for the development of predictive, preventive, and personalized medicine [2].

For every disease or phenotypic changes, some metabolites are upregulated and/or some are downregulated from standard within a cell. The metabolites which are upregulated or downregulated between disease and control groups are known as differentially expressed (DE) metabolites. The classic approach to identify differentially expressed metabolites is Student's *t*-test (for two classes of samples). If the pooled variance of two groups is small, then Student's *t*-test often increases the false discovery (FDR) for metabolic biomarker identification. However, biological fold-change (FC) approach is used to control the FDR [3]. Recently, volcano plot [4] is using to identify differentially expressed metabolites based on both *p* values from *t*-test and fold-change (FC) values [5]. In most of the cases, we get a large number of DE metabolites; therefore, we need to identify the most influential feature/metabolites from the set of DE metabolites. The conventional approaches for ranking the influential metabolites are *p* value and fold-change approach. However, Gevrey et al. in 2003 [6] computed the contribution of features using artificial neural network model and also Rakotomamonjy (2003) [7] and Ishak and Ghattas (2005) [8] suggested a new method for variable/feature selection on ranking score derived from support vector machine (SVM). Thus, in this paper, we have used the state of the art supervised learning technique SVM for ranking the most influential metabolites (biomarkers) according to the importance. However, the performance of true biomarker discovery is very much influenced by missing value imputation techniques [9] as well as outliers handling [10]. It is well known that mass spectrometry (MS) based metabolomics dataset frequently contains missing values [11–13] and often contains outliers [14] due to several reasons, like experimental, analytical, computational, and biological hazard. Although several missing imputation techniques are available for metabolomics data analysis like Zero imputation [11], mean imputation [11], median imputation [11], kNN imputation [15], RF imputation [16], missing value replaced by half of the minimum value found in each metabolite [17], replacing missing values by probabilistic principal component analysis (PPCA) [18], Bayesian PCA (BPCA) [9, 19], multiple imputation with expectation maximization (EM) algorithm, and Monte Carlo Markov chain (MCMC) method [20], and so on, all the above methods can only solve the missing imputation problem. However, these missing imputation techniques are sensitive to outliers and cannot handle the outliers problem simultaneously because these methods did not implement any robust function or outliers detection and replacement algorithms directly. Moreover, the published outliers handling method does not deal with missing values.

Therefore, in this paper we have developed a new robust technique for biomarker identification using the groupwise RSVD [21], *t*-test, FC analysis, and SVM based feature selection approaches that can identify biomarker correctly by imputing missing values and solving outliers problem simultaneously.

To compare the performance of the proposed biomarker identification technique with those of the conventional techniques, we considered three well known traditional and recently used biomarker identification techniques (missing imputation techniques with *t*-test and FC values): Zero imputation with *t*-test and FC (Zero + *t* + FC), kNN imputation with with *t*-test and FC (kNN + *t* + FC), and RF imputation with *t*-test and FC (RF + *t* + FC).

## 2. Materials and Methods

In this paper, we have proposed a robust technique to identify metabolomic biomarker that can handle missing values as well as outliers problem at the same time. To investigate the performance of the proposed method, we considered existing three popular missing value imputation techniques, Zero imputation, kNN imputation, and RF imputation, and also used *t*-test and fold-change approach for biomarker identification. In Zero imputation, all the missing values of a dataset were replaced by Zero. In kNN imputation [15], missing value was computed by averaging of nonmissing values of its first *k* number of nearest neighbours. In R platform kNN imputation method can be found from the library “*impute.*” Missing imputation algorithm through random forest [16] is a tree based regression and classification technique which is suitable for both parametric and nonparametric dataset [22]. In R-language this method can be found from the library “*missForest.*” The short description of the proposed biomarker identification technique is given below.

### 2.1. Biomarker Identification Technique in Presence of Outliers and Missing Values (Proposed)

Let us consider a metabolomics data matrix *X* = (*x*_*ij*_), *i* = 1,2,…, *p* and *j* = 1,2,…, *n*, with *p* metabolites and *n* samples that contains missing values and outliers, where the rows of *X* represent the different metabolites and the columns of *X* represent the different samples. In metabolomics data analysis, we can talk about biomarker identification whenever a metabolite or a set of metabolites are differentially expressed between two groups (disease and control) of samples in a metabolomics dataset. In that case, the metabolomics dataset can be partitioned according to the groups of samples as

(1) X=x11x12⋯x1g1x21x22⋯x2g1⋮⋮⋱⋮xp1xp2⋯xpg1︷group-1 x1g1+1x1g1+2⋯x1nx2g1+1x2g1+2⋯x2n⋮⋮⋱⋮xpg1+1xpg1+2⋯xpn︷group-1,

where *g*_1_ is the number of subjects of group-1 and (*n* − *g*_1_) is the number of subjects of group-2.

In this paper, we have proposed a new RSVD based metabolomic biomarker identification technique in presence of missing values and outliers. The RSVD of a data matrix *X* can be written as *X* = *U*Λ*V*^*T*^, where *U* is a column orthonormal matrix, *V* is a row orthogonal matrix, and Λ is a diagonal matrix that contains the singular values. *U* and *V* contain the eigen vectors of *XX*^*T*^ and *X*^*T*^*X*, respectively. If we want to approximate *X* by $\tilde{X}$ using rank *r* then we can write $\tilde{X}$ as $\tilde{X}=\lambda_{1}u_{1}v_{1}^{T}+\lambda_{2}u_{2}v_{2}^{T}+\cdots +\lambda_{r}u_{r}v_{r}^{T}$. The number of *r* is selected in such a way that first *r* number of *λ*'s can explain at least (1 − *α*)100% of total variation of data, where the value of *α* depends on user interest. The steps of the metabolomic biomarker identification algorithm are given below.

**Step 1 .**

Partition the *X* matrix as *X* = (*X*_1_*X*_2_) according to disease and control group of samples, where

(2) X1=x11x12⋯x1g1x21x22⋯x2g1⋮⋮⋱⋮xp1xp2⋯xpg1,X2=x1g1+1x1g1+2⋯x1nx2g1+1x2g1+2⋯x2n⋮⋮⋱⋮xpg1+1xpg1+2⋯xpn.

**Step 2 .**

Apply the RSVD for each partitioned matrix *X*_1_, *X*_2_ and approximate those as ${\tilde{X}}_{1},{\tilde{X}}_{2}$ by rank *r*, where *r* is selected in such a way that first* r* number of *λ*'s can explain at least (1 − *α*)100% of total variation of data, where the value of *α* depends on user interest; in our case we choose *α* = 0.05.

**Step 3 .**

Reconstruct each partitioned data matrix as ${\hat{X}}_{1},{\hat{X}}_{2}$ by replacing the missing values and outlying cells of *X*_1_, *X*_2_ using the corresponding values of the approximated data matrices ${\tilde{X}}_{1},{\tilde{X}}_{2}$, respectively, where outlier is detected by using outlier detection rules like interquartile range (IQR) rule [23].

**Step 4 .**

Reconstruct the metabolomics data matrix *X* as $\hat{X}=\left({\hat{X}}_{1}{\hat{X}}_{2}\right)$.

**Step 5 .**

Compute the differentially expressed metabolite from the reconstructed metabolomics data matrix $\hat{X}$ using *p* value and FC value. The *p* value can be calculated using *t*-test. To declare the metabolite as differentially expressed, choose the threshold *p* value using Bonferroni correction and absolute fold-change (FC) cut-offs of >2.

**Step 6 .**

Rank the differentially expressed metabolites according to their influence or importance using support vector machine (SVM) [24].

**Step 7 .**

List the first few top upregulated and downregulated metabolites from Step 6 that classify the samples with higher accuracy and declare those metabolites as biomarker. Upregulated and downregulated metabolites can be identified by the sign of fold-change values.

The R-code of the proposed method can be found in the following URL: http://www.statru.org/wp-content/uploads/2010/12/MetabBio.zip

### 2.2. Dataset Description

Both simulated and real metabolomics datasets have been used to demonstrate the performance of the proposed method in a comparison of the other methods.

#### 2.2.1. Simulated Dataset

We have simulated metabolomics datasets using the one-way ANOVA model, defined by *y*_*ijk*_ = *μ*_*i*_ + *g*_*ij*_ + ∈_*ijk*_, where *y*_*ijk*_ is the intensity of *i*th metabolite, *j*th group, and *k*th sample; *μ*_*i*_ denote the average intensity of metabolite *i*; *g*_*ij*_ is the *j*th group effect for *i*th metabolite and ∈_*ijk*_ is the random error term. In this linear model, we have taken *μ*_*i*_*～uniform *(4, 8) and ∈_*ijk*_*～N*(0,1). We have also taken two types of metabolites: (i) differentially expressed (DE) metabolites and (ii) equally expressed (EE) metabolites. DE metabolites are divided into two groups: upregulated metabolites and downregulated metabolites. Disease and control group effects for upregulated metabolites are *g*_*ij*_ = *N*(2,1) and *g*_*ij*_ = *N*(0,1); for downregulated metabolites disease and control group effects are *g*_*ij*_ = *N*(0,1) and *g*_*ij*_ = *N*(2,1) and in case of equally expressed metabolites the group effect for both cases is *g*_*ij*_ = *N*(0,1). To make a simulated metabolomics dataset, we have taken 500 metabolites (30 DE metabolites and 470 EE metabolites) and 80 samples (45 control and 35 disease). From 500 metabolites, the first 15 metabolites have been included as upregulated metabolites and 121 to 135 metabolites have been incorporated as downregulated metabolites for disease samples. We have also included different rates of missing values (10%, 15%, and 20%) in this dataset, where 50% missing were given at random and the rest of the missing were given for lower values. To measure the performance of the proposed method in different condition, we have incorporated different rates (3%, 5%, 7%, 10%, and 15%) of outliers in simulated dataset. The outliers in the *i*th metabolite were taken from normal distribution with mean = 5*∗μ*_*i*_ and variance = *σ*_*i*_^2^, where *μ*_*i*_ and *σ*_*i*_^2^ are the mean and variance of the *i*th metabolite. We have distributed the outliers randomly by different rates (3%, 5%, 7%, 10%, and 15%) in the data matrix cell; therefore, outliers may fall anywhere in the data matrix. We simulated 100 datasets using the above conditions and also measured the performance of the proposed method using these simulated dataset.

#### 2.2.2. Real Metabolomics Dataset

In this paper, we have used a publicly available real metabolomics dataset on the metabolomic effect of hepatocellular carcinoma (HCC) that contains the abundance level measurements of metabolites from different subjects. This dataset was originally produced by Patterson et al. [25]. To extract the metabolomic profile from the sample, ultra-performance liquid chromatography coupled with electrospray ionization/quadrupole-time-of-flight mass spectrometry (UPLC-ESI-Q-TOF-MS) was used and this dataset was normalized by Pareto scaling. This data matrix contains 1388 rows and 57 columns. Each row is a metabolite detected by the retention time (rt) and mass to charge (*m*/*z*) ratio that were included in first column and the second column, respectively. The remaining 55 columns were different subjects that came from two groups. 20 subjects were from the hepatocellular carcinoma (HCC) group and 35 subjects were from the control group. There were 26.52% missing values/cells in this dataset. More details about the data can be found in the article of Patterson et al. [25]. To measure the performance of the proposed method, we calculated the error between original and reconstructed data that we can understand how well the proposed method reconstructs the data. We also modified the real dataset by replacing 5% existing values as missing and changing 5%, 10%, and 15% real values by *N*(5*∗μ*_*i*_, *σ*_*i*_^2^), where *μ*_*i*_ and *σ*_*i*_^2^ are the mean and variance of the *i*th metabolite in the HCC data matrix.

### 2.3. Performance Measurement Criteria

To investigate the performance of the proposed method, we calculated the root mean square error (RMSE) between the original dataset and reconstructed dataset that we can easily identify better method to reconstruct the data. The formula of RMSE is $RMSE=\sqrt{(1/np)\sum_{i=1}^{p}\sum_{j=1}^{n}{\left(x_{ij}-{\hat{x}}_{ij}\right)}^{2}}$, where *x*_*ij*_ and ${\hat{x}}_{ij}$ are the original and reconstructed value of the *i*th row and *j*th column of the data matrix, respectively.

For simulated dataset, we also assess the performance of the proposed biomarker identification technique compared to the other techniques where we consider three performance indices: (i) ROC curve, (ii) area under the ROC curve (AUC), and (iii) misclassification error rate (MER). To calculate the above measures, first we identify the DE metabolites using four different techniques including the proposed technique. Using the above results, we draw ROC curve using true positive rate (TPR) and false positive rate (FPR). In a binary classification system if a positive instance is correctly classified as positive, it is called true positive (TP); however, if it is wrongly classified as negative then it is called false negative (FN). If a negative instance is correctly classified as negative, it is called true negative (TN); otherwise, it is called false positive (FP). The TPR and FPR are calculated by

(3) TPRnumber  of  correctly  classified  as  positiveTotal  number  of  positives=nTPnTP+nFNFPRnumber  of  wrongly  classified  as  positiveTotal  number  of  negatives=nFPnTN+nFP.

ROC curve plots contain the TPR in *y*-axis and FPR in *x*-axis.

In real metabolomics dataset, we do not know the class of metabolites; therefore, we measure the performance of the proposed biomarker identification method in a comparison of the other considering methods by sample classification using SVM classifier on the basis of the computed DE metabolites. Using the sample classification results, we calculate the performance indices: ROC curve, accuracy, sensitivity, specificity, positive predicted value, negative predicted value, and balanced accuracy.

A better method would produce smaller values for FPR and MER and larger values for TPR and area under the ROC curve (AUC) by any DE metabolite detector.

## 3. Results and Discussion

We have evaluated the performance of the proposed method using both simulated and real data (hepatocellular carcinoma) analysis results. The detailed description of the simulated and real data analysis results is discussed in Sections 3.1 and 3.2.

### 3.1. Simulated Data Analysis Results

To investigate the performance of the proposed method for imputing missing values, we calculated the average RMSE between original and reconstructed data matrix using 100 simulated datasets with different rates (10%, 15%, and 20%) of missing values in both absence and presence of outliers. The average RMSE for different methods in both different rates (10%, 15%, and 20%) of missing values and presence of different rates (0%, 3%, 5%, 7%, 10%, and 15%) of outliers have been given in Table 1. Table 1 showed that in absence of outliers all the methods except Zero imputation produce similar as well as smaller average RMSE between original and reconstructed data matrices; however, in presence of outliers, the proposed method gave the smaller average RMSE between original and reconstructed data matrices compared to other (Zero, kNN, and Zero imputation) techniques. Therefore, we could say that the proposed method reconstructs the simulated data very closely in presence of both different rates (0%, 3%, 7%, 10%, and 15%) of outliers and different rates (10%, 15%, and 20%) of missing values.

**Figure 1:**
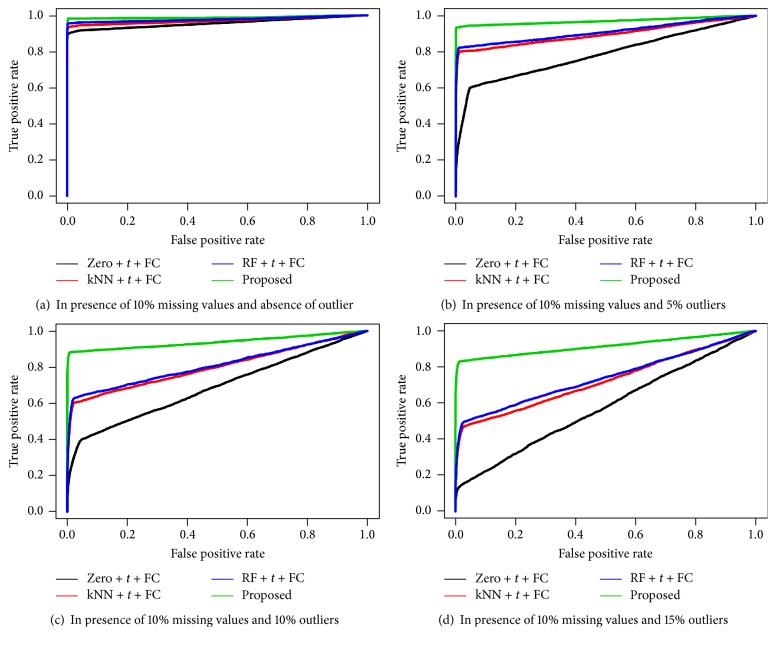
Performance investigation of different techniques using ROC curve for simulated data in both presence of 10% missing values and different rates of outliers (0% (a), 5% (b), 10% (c), and 15% (d)).

**Figure 2:**
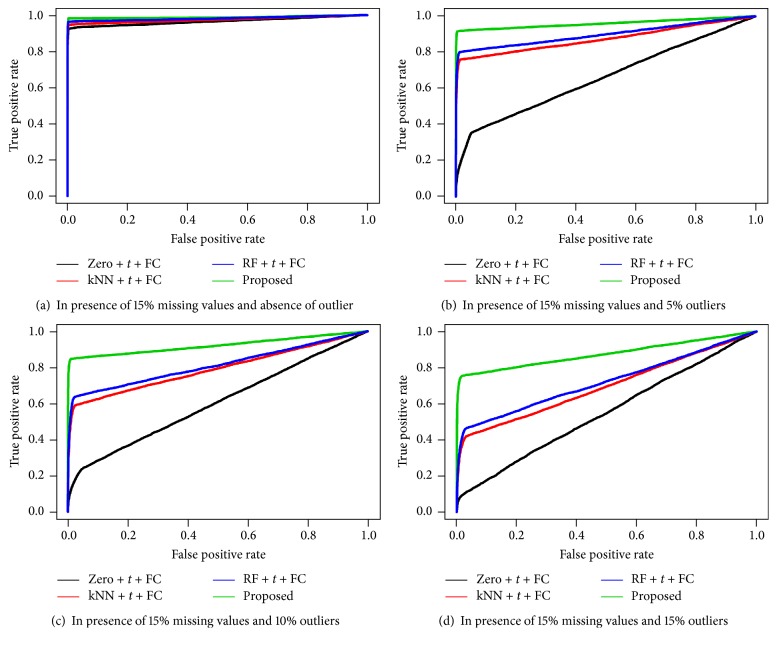
Performance investigation of different techniques using ROC curve for simulated data in both presence of 15% missing values and different rates of outliers (0% (a), 5% (b), 10% (c), and 15% (d)).

**Figure 3:**
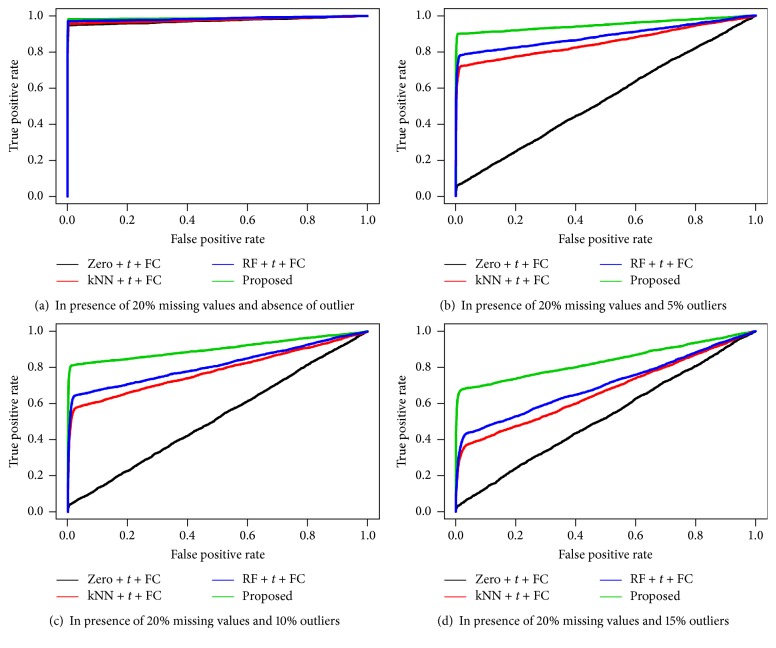
Performance investigation of different techniques using ROC curve for simulated data in both presence of 20% missing values and different rates of outliers (0% (a), 5% (b), 10% (c), and 15% (d)).

We also performed simulation studies to measure the performance of the four different techniques including our proposed technique for identifying DE and EE metabolites. To measure the performance, first we identified the DE and EE metabolites from the simulated dataset using four different techniques (Zero + *t* + FC, kNN + *t* + FC, RF + *t* + FC, and Proposed) in both presence of 10%, 15%, and 20% missing values and different rates (0%, 5%, 10%, and 15%) of outliers. In case of simulated dataset the metabolites classes were known; therefore, using the given metabolites classes and predicted metabolites classes, we calculated the three different performance indices, ROC curve, AUC values, and MER, for each biomarker identification technique. We repeated the above computations for 100 simulated datasets and calculated the average of each performance measure and also presented them in Figures 1, 2, and 3 and Table 2. From Figures 1, 2, and 3, it is seen that the proposed biomarker identification technique produced larger average TPR regarding any point of average FPR compared to the other techniques. Also from Table 2, we can observe that the proposed biomarker identification technique gave larger average AUC values and smaller average MER compared to other (Zero + *t* + FC, kNN + *t* + FC, and RF + *t* + FC) techniques in both absence and presence of outliers for different percentage (10%, 15%, and 20%) of missing datasets. Therefore, we could conclude that the proposed biomarker identification technique is better among the four techniques in both presence of missing values and different rates of outliers.

### 3.2. Real HCC Data Analysis Results

To measure the performance of the proposed method in a comparison of the other methods for imputing missing values in presence of outliers, we modified 100 real datasets by replacing 5% existing cell as missing and changing 5%, 10%, and 15% existing cell by (5*∗μ*_*i*_, *σ*_*i*_^2^), where *μ*_*i*_ and *σ*_*i*_^2^ are the mean and variance of the *i*th metabolite in the HCC data matrix and also calculated the average RMSE between real dataset and reconstructed datasets. The average RMSE for different missing imputation techniques including our proposed one have been given in Table 3. We can observe from Table 3 that the proposed technique has produced smaller average RMSE in all the cases; that is, the proposed technique could reconstruct the real data more closely compared to the other methods in presence of different rates of outliers.

**Figure 4:**
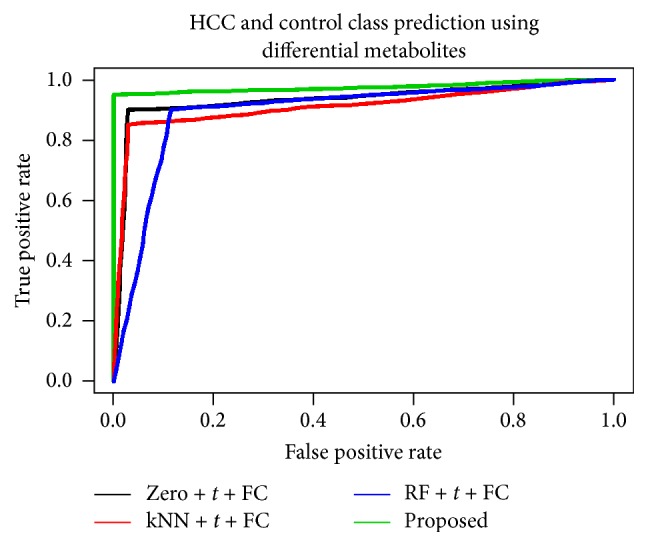
Performance investigation of different techniques using ROC curve for real HCC dataset.

To identify the robust method for biomarker identification technique using real dataset, we also computed the different performance indices, ROC curve, accuracy, sensitivity, specificity, positive predicted value, negative predicted value, and balanced accuracy for sample classification using the set of DE metabolites. To compute the performance indices, we identified the set of DE metabolites by four different biomarker identification techniques including the proposed one and using the identified DE metabolites, we classified the sample by SVM classifier. We used the fivefold cross validation to compute the performance indices whose results have been given in Figure 4 and Table 4.

**Figure 5:**
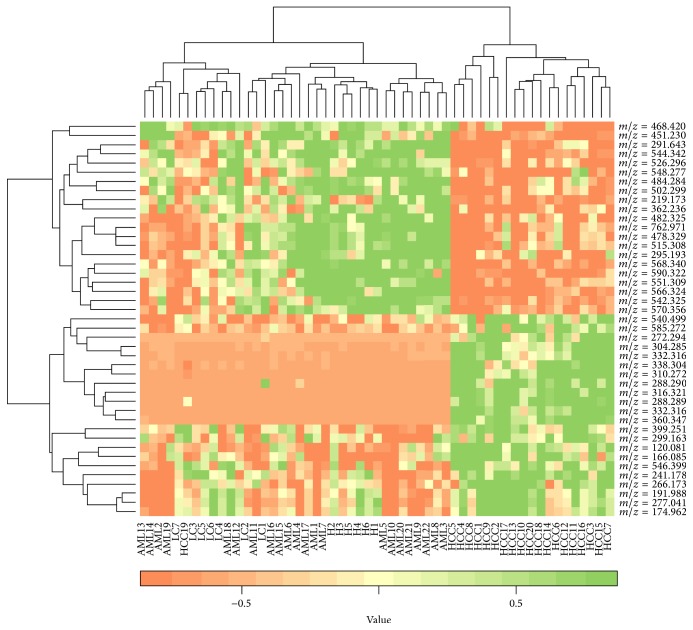
Mining 43 differentially expressed (DE) metabolites identified by the proposed method.

**Figure 6:**
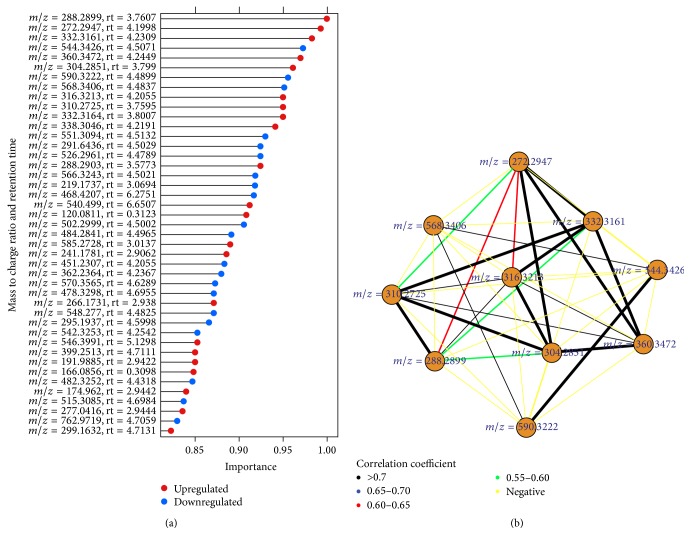
Ranking the 43 differentially expressed (DE) metabolites according to their importance (a) and correlation network of top ten ranked DE metabolites (b).

All performance indices of Figure 4 and Table 4 showed that the proposed biomarker identification technique is better among the four techniques. Therefore, we identified the differentially expressed metabolites using the proposed biomarker identification technique and categorized the upregulated and downregulated metabolites using the value of the FC as well as mining the DE metabolites using heatmap plot and hierarchical clustering technique in Figure 5. We also ranked the 43 DE metabolites using SVM technique according to their importance. The ranked upregulated and downregulated metabolites have been showed in Figure 6(a). From Figure 5, it was clear that the identified 43 DE metabolites can correctly and clearly cluster the samples into two groups: control group and HCC group. We could also observe that, from the 43 DE metabolites, 21 metabolites were downregulated and the remaining 22 metabolites were upregulated in HCC patients. In Figure 6(a) red circles indicated upregulated metabolites and blue circles indicated downregulated metabolites. From Figure 6(a), we could say that the metabolite with *m*/*z* = 288.2899, rt = 3.7607 is the most influential upregulated metabolite for HCC disease. We got 7 upregulated and 3 downregulated metabolites from the top ten ranked DE metabolites and we also drew the correlation network of the top ten ranked DE metabolites (see Figure 6(b)). Thus on the basis of Figure 6, we could take the top four upregulated metabolites (“*m*/*z* = 288.2899, rt = 3.7607,” “*m*/*z* = 272.2947, rt = 4.1998,” “*m*/*z* = 332.3161, rt = 4.2309,” and “*m*/*z* = 360.3472, rt = 4.2449”) and the top two downregulated metabolites (“*m*/*z* = 544.3426, rt = 4.5071” and “*m*/*z* = 590.3222, rt = 4.4899”) as a set of biomarkers for HCC disease. We also observed that, for sample classification, this set of biomarkers (six metabolites) gave 100% classification accuracy in fivefold cross validation. However, for taking a final decision to declare as a set of biomarkers, further wet laboratory experimental validation is required.

## 4. Conclusion

We have shown that missing values and outliers play important role in different biomarker identification techniques to identify biomarkers from GC-MS metabolomics data. We have also shown that performance of existing traditional biomarker identification procedure is very much influenced by outlying observations and missing values. Therefore, in this paper, we have proposed a new biomarker identification procedure using groupwise robust singular value decomposition, *t*-test, and FC approach. We investigated the performance of the proposed method in a comparison of traditional methods using both simulated and real data analysis. On the basis of our computational findings, we could conclude that the proposed method is better performer than the traditional techniques in both absence and presence of outliers. Therefore, our suggestion is to use the proposed biomarker identification procedure for metabolomic biomarker identification in GC-MS metabolomics data. Using the proposed biomarker identification technique, we have got four upregulated and two downregulated metabolites for hepatocellular carcinoma disease and further research can be the wet laboratory experimental validation to take a final decision.

## Figures and Tables

**Table 1:** Performance measurement of different methods using average RMSE between original and reconstructed data matrix for different percentage of missing values in absence and presence of outliers using simulated datasets.

Methods with different conditions		Without outliers	3% outliers	5% outliers	7% outliers	10% outliers	15% outliers
For 10% missing values	Zero	1.56	1.92	2.10	2.35	2.48	2.69
	kNN	**0.77**	1.42	1.71	2.05	2.23	2.43
	RF	0.82	1.45	1.73	2.07	2.24	2.45
	Proposed	**0.77**	**1.15**	**1.19**	**1.24**	**1.37**	**1.48**

For 15% missing values	Zero	2.19	2.42	2.56	2.72	2.91	3.22
	kNN	**0.91**	1.47	1.77	2.05	2.36	2.51
	RF	0.97	1.51	1.80	2.08	2.39	2.52
	Proposed	0.92	**1.24**	**1.28**	**1.34**	**1.50**	**1.64**

For 20% missing values	Zero	2.51	2.71	2.88	2.96	3.14	3.37
	kNN	**1.00**	1.58	1.94	2.12	2.45	2.69
	RF	1.07	1.63	1.98	2.15	2.48	2.75
	Proposed	1.01	**1.29**	**1.34**	**1.41**	**1.59**	**1.71**

**Table 2:** Performance measurement of different methods using average AUC values and MER (in parentheses) for different percentage of missing values in absence and presence of outliers.

Different conditions	Proposed	Zero + *t* + FC	kNN + *t* + FC	RF + *t* + FC
10% missing values and absence of outlier	**99.14 (0.86)**	95.72 (4.28)	97.82 (2.18)	98.58 (1.42)
10% missing values and 5% outliers	**96.86 (3.14)**	71.19 (28.81)	85.53 (14.47)	86.25 (13.75)
10% missing values and 10% outliers	**94.25 (5.75)**	65.69 (34.31)	79.08 (20.92)	80.10 (19.9)
10% missing values and 15% outliers	**91.75 (8.25)**	57.69 (42.31)	73.14 (26.86)	74.33 (25.67)
15% missing values and absence of outlier	**98.93 (1.07)**	95.07 (4.93)	97.72 (2.28)	97.98 (2.02)
15% missing values and 5% outliers	**95.46 (4.54)**	63.29 (36.71)	84.10 (15.9)	85.14 (14.86)
15% missing values and 10% outliers	**92.67 (7.33)**	59.24 (40.76)	78.07 (21.93)	79.89 (20.11)
15% missing values and 15% outliers	**87.49 (12.51)**	56.16 (43.84)	70.13 (29.87)	72.46 (27.54)
20% missing values and absence of outlier	**98.84 (1.16)**	94.44 (5.56)	97.44 (2.56)	97.47 (2.53)
20% missing values and 5% outliers	**94.89 (5.11)**	54.27 (45.73)	82.46 (17.54)	84.81 (15.19)
20% missing values and 10% outliers	**90.44 (9.56)**	53.71 (46.29)	77.07 (22.93)	79.48 (20.52)
20% missing values and 15% outliers	**83.47 (16.53)**	54.11 (45.89)	67.69 (32.31)	70.70 (29.30)

**Table 3:** Performance measurement of different methods using average RMSE between original and reconstructed data matrix for real dataset by additionally imputing 5% missing values and different rates (5%, 10%, and 15%) of outliers.

Methods	5% outliers	10% outliers	15% outliers
Zero	22.76	29.98	41.48
kNN	19.38	27.47	37.81
RF	17.15	26.05	35.29
Proposed	**8.41**	**12.35**	**17.93**

**Table 4:** Performance investigation of different techniques by sample classification using DE metabolites.

Different techniques	Accuracy (%)	Sensitivity (%)	Specificity (%)	Positive predicted value (%)	Negative predicted value (%)	Balanced accuracy (%)
Zero + *t* + FC	94.55	97.14	90.00	94.44	94.74	93.57
kNN + *t* + FC	92.73	97.14	85.00	91.89	94.44	91.07
RF + *t* + FC	89.09	88.57	90.00	93.94	81.82	89.29
Proposed	**98.18**	**100.00**	**95.00**	**97.22**	**100.00**	**97.50**
